# Prevalence of medial tibial stress syndrome in the British Armed Forces: a population-based study

**DOI:** 10.1136/military-2024-002788

**Published:** 2024-11-21

**Authors:** Emma Farquharson, A J Roberts, A I Warland, N Parnis, N E O’Connell

**Affiliations:** 1 Department of Health Sciences, Brunel University London, Greater London, UK; 2 Academic Department of Military Rehabilitation, DMRC Stanford Hall, Loughborough, UK; 3 Army Recruit Health and Performance Research, Medical Branch, HQ Army Recruiting and Initial Training Command, Ministry of Defence, Upavon, UK; 4 Department of Health Sciences, Centre for Wellbeing Across the Lifecourse, Brunel University London, Greater London, UK; 5 Academic Skills Team, Brunel University London, Greater London, UK

**Keywords:** REHABILITATION MEDICINE, EPIDEMIOLOGY, Musculoskeletal disorders

## Abstract

**Introduction:**

Medial tibial stress syndrome (MTSS) is common in the Armed Forces due to the physical demands placed on service personnel (SP). There are no large studies reporting the extent to which MTSS affects the Armed Forces. A retrospective cross-sectional study design was used to report the annual prevalence of MTSS in the British Armed Forces and in training units and healthcare utilisation.

**Methods:**

Secondary data were sourced from the electronic medical records for all SP with MTSS (20 257) between 1 January 2010 and 31 December 2018. Prevalence was calculated annually across the Armed Forces and in recruits. Healthcare utilisation (number of contacts and days under the care of a healthcare professional) was reported according to characteristics of SP (sex, age, ethnicity, service branch, body composition measurement and medical discharge).

**Results:**

Over 9 years, 20 257 SP were seen for MTSS. Prevalence of MTSS decreased across the Armed Forces, from 2.19% (95% CI 2.12 to 2.26) in 2013 to 1.61% (95% CI 1.55 to 1.68) in 2018. The prevalence of MTSS was 2.7 times higher in recruits, affecting 4.34% (95% CI 4.00 to 4.69) in 2018. In 2018, the prevalence in female recruits was over four times higher (7.03%, 95% CI 5.74 to 8.32) than trained female SP (1.60%, 95% CI 1.39 to 1.81) and higher than male recruits (4%, 95% CI 3.65 to 4.35). Comparing service branches, royal marines had the least healthcare input (median contacts (IQR): 3 (1–7.5)) over the least number of days (median days (IQR): 17 (0–154)), with the royal air force receiving the most (median contacts (IQR): 5 (2–13)) over the greatest number of days (median days (IQR): 76 (4–349)).

**Conclusion:**

The prevalence of MTSS has reduced; however, it remains high in subsections of the Armed Forces, particularly in female recruits. There is a large variation in the amount and duration of healthcare input SP received for MTSS.

**WHAT IS ALREADY KNOWN ON THIS TOPIC**

- Medial tibial stress syndrome is common in military service personnel and athletes who are highly active, with slow recovery times.
- Prevalence studies to date are based on small populations, for example, recruits and specific athletic populations. The prevalence of MTSS across the UK Armed Forces population prior to our study was not known.

**WHAT THIS STUDY ADDS**

- Our study found that MTSS affected approximately 1.61% of all service personnel and 4.34% of recruits in the British Armed Forces in 2018.
- The prevalence of MTSS is highest in female recruits, affecting 7.03% of female recruits in the British Armed Forces in 2018.

**HOW THIS STUDY MIGHT AFFECT RESEARCH, PRACTICE OR POLICY**

- Having a better understanding of MTSS can help inform policy and guideline development, clinical priorities and future health economic models.

## Introduction

 Medial tibial stress syndrome (MTSS) is a musculoskeletal injury common to athletes^1^ and Armed Forces personnel.^2^ It presents as pain along the posterior-medial border of the tibia that occurs with physical loadings, such as running or marching, and a diagnosis is made when other forms of leg pain have been excluded.^3^

In the UK Armed Forces, musculoskeletal injury, including MTSS, is the leading cause of medical discharge, which can result when service personnel (SP) are unable to perform their duties and alternative employment is not available. In the British Army, 57% of all medical discharges between March 2017 and April 2018 were because of musculoskeletal injuries.^4^ MTSS was reported to be the second most common injury in those presenting to their medical centre during a 26-week training programme, accounting for 5.67% of suspected injuries and the largest number of training days lost (19.8%) due to injury.^5^ To date, the highest reported incidence of MTSS was in Australian Naval recruits during a 10-week basic training programme, where 35% were diagnosed with the syndrome.^3^ The impact of MTSS on SP and the Armed Forces as an employer should not be underestimated. Recovery times can take on average 4–5 months^6^ and in those who have previously had MTSS, there is a higher risk of recurrence of symptoms.^7^ For SP with MTSS, managing the syndrome can impact training and deployment, take time away from work to attend medical appointments and rehabilitation and for some can lead to medical discharge from the forces. The psychological impact of being injured in the Armed Forces can lead to fear of the injury affecting future career opportunities and lead to negative perceptions (weakness or ineptitude) associated with injury.^8^ The cost of MTSS to the Armed Forces is also likely to be high, not only financially for the medical and rehabilitation costs but also operationally, with musculoskeletal injury affecting the forces’ ability to maintain an effective deployable workforce.

To date, only small-scale studies have reported the prevalence of MTSS, often in specialised populations, for example, military recruits^3^ or athletes.^9^ Determining the prevalence of MTSS across the entire Armed Forces, which had approximately 150 000 SP in 2018 alone, will help better understand the syndrome and inform policy and guideline development, clinical priorities and future health economic models.

The primary aim of our study is to report the prevalence of MTSS across the entire British Armed Forces and in recruits between 2013 and 2018. A secondary aim is to report the healthcare utilisation for MTSS, including the number of calendar days spent under the care of healthcare professionals and the number of contacts with healthcare professionals. Further objectives are to present the percentage of SP with MTSS who have been discharged from the Armed Forces with lower limb injuries. The characteristics of SP with MTSS will also be reported (eg, sex, ethnicity, service branch, age when first seen for MTSS, etc).

## Methods

A retrospective cross-sectional study design was used, sourcing secondary data from the Defence Medical Information Capability Programme (DMICP; the medical electronic notes system used in the UK Armed Forces), for related records entered between 1 January 2010 and 31 December 2018.

The DMICP is used by all healthcare professionals across the British Armed Forces, including consultants, medical officers, physiotherapists, nurses, occupational therapists and podiatrists. All SP medical records are stored on DMICP from the point of joining the Forces until the point that they leave. Defence Statistics (a unit within the Armed Forces that compiles and provides professional analytical, economic and statistical services and advice to the Armed Forces and external bodies) provided a database for all SP that had contact with a healthcare professional for MTSS. Every encounter SP has with a healthcare professional is recorded on DMICP and coded using a diagnostic read code, determined by the clinician entering the notes. The MTSS database included all entries linked to a read code related to MTSS (see online supplemental figure 1) or exertional lower limb pain (ELLP), as MTSS can also be coded under this umbrella term. Exertional lower leg pain encompasses several conditions, including chronic exertional compartment syndrome, popliteal entrapment and MTSS. With MTSS being by far the most prevalent of the syndromes, it was decided to include ELLP, as not including ELLP would lead to many with MTSS being missed rather than added if included. Gurkhas (soldiers recruited from Nepal), all regular serving personnel (trained) and those in training (ie, recruits) but not reservists were included.

The MTSS database contained each contact SP with MTSS had with a healthcare professional, whether they were seen for assessment, treatment or administrative purposes. Some SP had only one entry, while others had several entries spanning years. Data collected for each entry included sex, ethnicity, service branch, age and body composition measurement (at the time of the contact, BCM: body mass index and waist circumference measurement to identify an individual’s level of health risk—full details in online supplemental file 1). Data collected on SP with MTSS was compared with that of all Armed Forces SP based on published data from the Biannual Diversity Statistics and the Quarterly SP Statistics.^10^ Whether SP had been medically discharged from the military for a lower-limb injury was also reported, including whether the injury was a principal (main medical cause of the discharge) or contributory (any other conditions identified that would result in a medical discharge) factor.

The annual prevalence of MTSS across the British Armed Forces was calculated between 2013 and 2018 based on sex, service and training status (trained or untrained, ie, recruits). Baseline data (total number of SP in the Armed Forces, within each sex, service, untrained and trained) were obtained via a freedom of information request to Ministry Of Defence Tri-Service Analysis. Although the MTSS database was available in 2010, baseline data were only available from 2013 to 2018. To improve consistency in reporting, prevalence is only reported from 2013 onwards. To calculate annual prevalence, the MTSS database was first divided into the respective years, with only the first entry SP had during that year being used in the analysis (to ensure each SP was only counted once). It should be noted that data reporting, whether trained or untrained, was dependent on the SP status at the time of the first entry in the medical record that year; that is, they may initially be untrained in previous years, then changed to trained in future years.

The way in which trained and untrained SP were reported in the baseline data changed during the study period. Trained SP was initially defined as SP that had completed phase 1 and phase 2 training. Phase 1 training includes all new entry training to provide basic military skills, and phase 2 training includes initial individual specialisation, subspecialisation and technical training. In October 2016, the army changed the definition of untrained to include only those who had not completed phase 1 training, where previously it included those who had not completed phase 2 training. To improve consistency in how prevalence was reported in this study, army SP in phase 1 and phase 2 training were reported as in training for the years 2017 and 2018, as per previous years. There was no change in how training was reported in the other services. For this paper, the terms recruits and untrained SP will be used interchangeably.

Descriptive statistics were used to describe the characteristics of those with MTSS and to explore the relationships between these characteristics. For example, the relationship between service/ethnicity and the number of contacts/lengths of time between the first and last contact. Missing data were assumed to be missing at random; therefore, pairwise deletion of cases was used. Missing data are reported for transparency.

## Results

### Prevalence of MTSS in the Armed Forces

**Figure 1:**
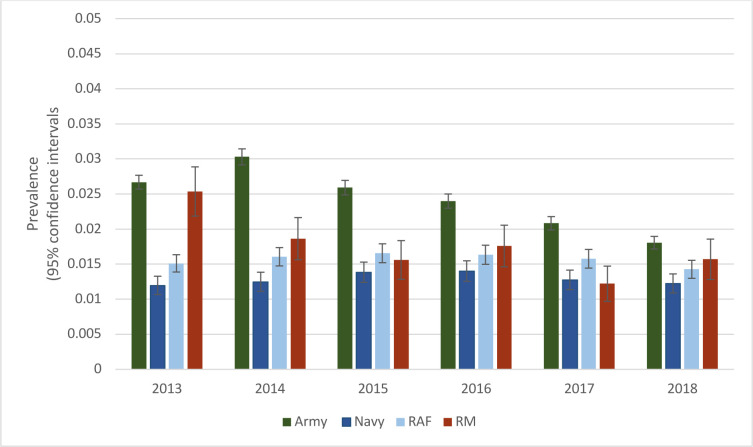
Prevalence of MTSS in each service in the Armed Forces (2013–2018). Error bars show the 95% CIs. MTSS, medial tibial stress syndrome.

The prevalence of MTSS across the Armed Forces was estimated to be 2.19% (95% CI 2.12 to 2.26) in 2013, which reduced to 1.61% (95% CI 1.55 to 1.68) in 2018. The prevalence of MTSS was higher in army SP than in all other services, with the lowest prevalence reported in navy SP (figure 1). Between 1 January 2010 and 31 December 2018, a total of 20 257 SP had 224 405 contacts with healthcare professionals for MTSS.

### MTSS in recruits

**Figure 2:**
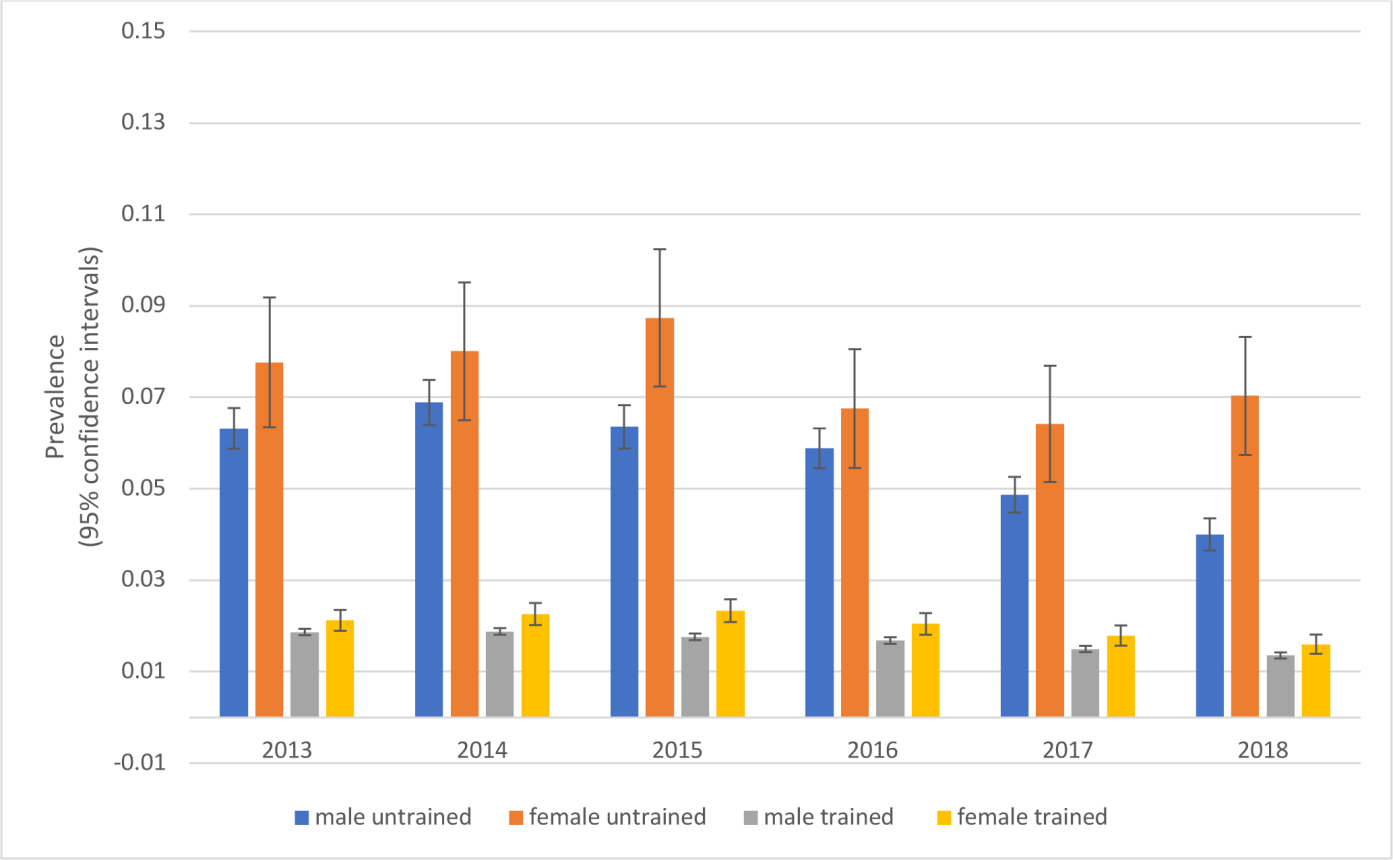
Prevalence of MTSS in female and male untrained and trained SP (2013–2018). Error bars show the 95% CIs. MTSS, medial tibial stress syndrome; SP, service personnel.

The prevalence of MTSS was more than double in recruits (untrained SP) compared with trained SP and higher still in female recruits throughout the period (figure 2).

**Figure 3:**
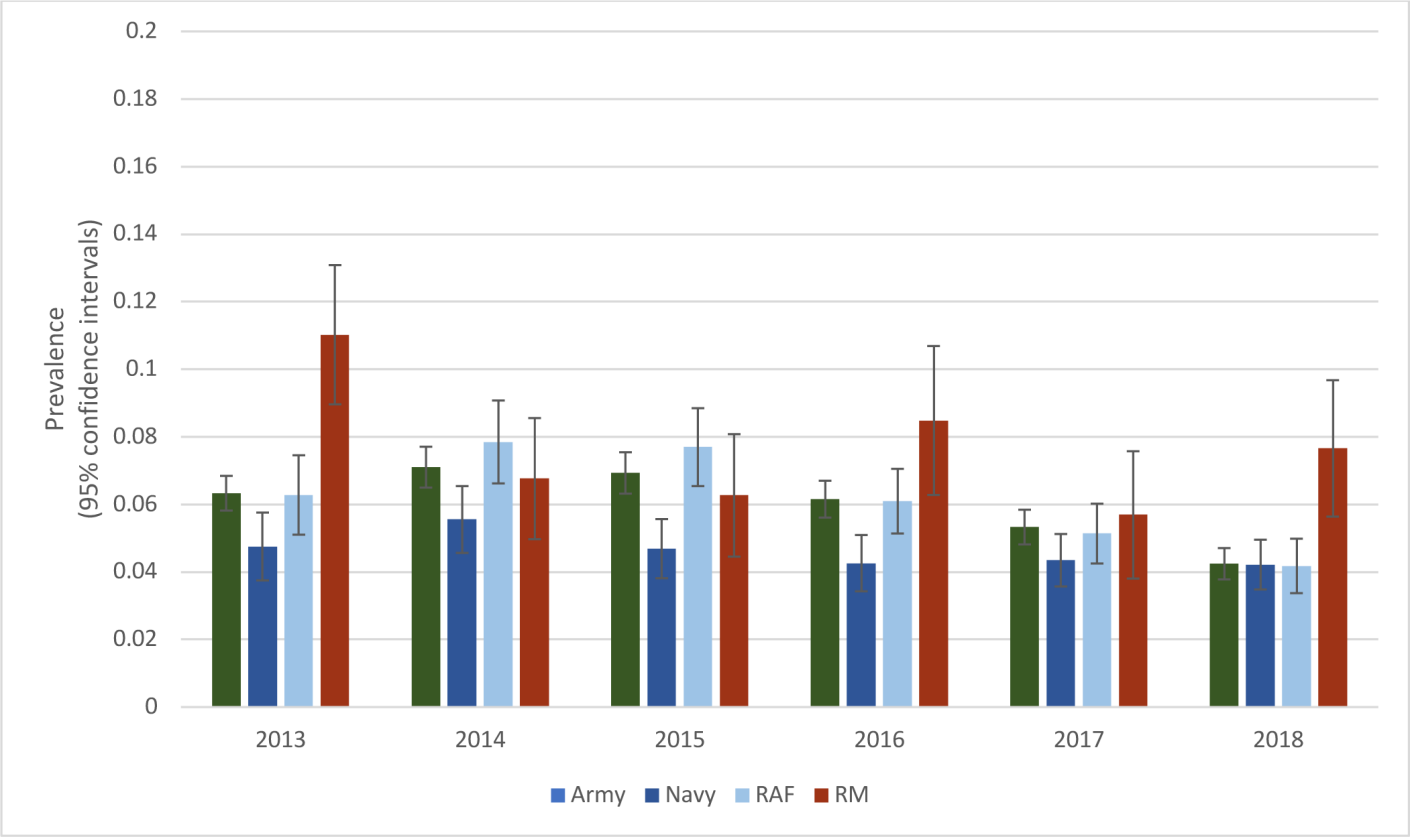
Prevalence of MTSS in recruits in each service (2013–2018). Error bars show the 95% CIs. MTSS, medial tibial stress syndrome.

The prevalence of MTSS in recruits varied annually between services (figure 3), with peaks demonstrated in royal marines (RM) (2013, 2016 and 2018). RM make up a smaller sample of the Armed Forces population and are more affected by small changes in MTSS numbers than other services.

### Characteristics of SP with MTSS

The median age when SP first had contact with a healthcare professional for MTSS was 24 (IQR 21–28) years, and the median length of service in the Armed Forces was 39 (IQR 10–88) months. The largest proportion of SP with MTSS were in the army, trained and had a normal body composition (table 1), consistent with the characteristics of most SP in the Armed Forces.

**Table 1:** Characteristics of SP with MTSS (1 January 2010 –31 December 2018)

Training status	Total number	Percentage
In training	6073	29.98%
Trained	13800:	68.12%
Unknown	384	1.90%
Service		
Army	13 921	68.72%
Navy	2036	10.05%
Royal Air Force	3259	16.09%
Royal Marines	1041	5.14%
BCM*		
Extreme risk	138	0.68%
Very high risk	485	2.39%
High risk	722	3.56%
Increased risk	1312	6.47%
No increased risk	9749	48.13%
Increased risk (under)	22	0.12%
Null	7829	38.65%

* BCM (see online supplemental information).

BCM, body composition measurement; MTSS, medial tibial stress syndrome; SP, service personnel.

Comparing the sex and ethnicity of personnel with MTSS to all SP in the Armed Forces demonstrated a higher percentage of female SP in the MTSS population (12.3%) compared with the percentage of female SP in the forces (9.93%). There was little difference in ethnicity between the two populations (table 2).

**Table 2:** Sex and ethnicity of SP

Sex	Mean annual number of SP with MTSS (mean %)	Mean annual number of SP in Armed Forces (mean %)	Mean annual percentage with MTSS (95% CI)
Female	393 (12.3%)	15 595 (9.93%)	2.52% (2.2 to 2.77)
Male	2798 (87.68%)	141 446 (90.07%)	1.98% (1.91 to 2.05)

Ethnicity	Mean annual number of SP with MTSS (mean %)	Mean annual number of SP in Armed Forces (mean %)	Mean annual percentage with MTSS (95% CI)
Black, Asian and minority ethnic	276 (8.65%)	13 943 (8.87%)	1.98% (1.75 to 2.21)
White	2893 (90.67%)	141 611 (90.11%)	2.04% (1.97 to 2.12)
Other	22 (0.68%)	1603 (1.02%)	1.37% (0.80 to 1.94)

With MTSS and in the Armed Forces (between 2013 and 2018).

MTSS, medial tibial stress syndrome; SP, service personnel.

### Medical discharge

Two per cent (Armed Forces=403, army=348, navy=17, royal air force (RAF)=14 and RM=24) of all SP with MTSS were medically discharged from the Armed Forces with a lower limb injury, where lower limb injury was either the principal or contributory cause of medical discharge. The rate of medical discharge was higher in recruits (3.4%, n=206).

### Healthcare input for MTSS in the Armed Forces

SP with MTSS had a median of four (IQR: 1–12) contacts with a healthcare professional, with almost a third (27%) only having one contact. The median number of days under the care of a healthcare professional for MTSS was 55 (IQR 0–306.5).

Female SP had one more contact and 11.5 more days under the care of a healthcare professional for MTSS than male SP. RM had fewer days in the care of a healthcare professional than all other services, and RAF personnel had more contacts and days in the care of a healthcare professional for MTSS than all other service branches (table 3).

**Table 3:** The median number of contacts and days in care according to service, sex, training status, ethnicity and BCM

	Number of contacts (median, IQR)	Days in care (median, IQR)
Service		
Army	4 (1–12)	57 (0–330)
Navy	3 (1–9)	31 (0–213.5)
Royal Air Force	5 (2–13)	76 (4–349)
Royal Marines	3 (1–7.5)	17 (0–154)
Sex		
Female	5 (2–14)	64.5 (1.00–332.5)
Male	4 (1–11)	53 (0–302)
Training status (unknown 1.90%)		
In training	4 (2–11)	33 (0–166)
Trained	4 (1–12)	70 (0–392)
Ethnicity (unknown 1.06%)		
Asian	3 (1–9)	22 (0–243.00)
Black	4 (1–12)	61 (0–490.00)
Other	4 (1–11)	54 (0–308.50)
White	4 (1–12)	55 (0–300.00)
BCM* (unknown: 38.65%)		
Extreme risk	5 (1–12.5)	127 (0–469.25)
High risk	5 (1–15)	94.5 (0–505.50)
Increased risk	4 (1–12)	69 (0–328.5)
Increased risk (under)	3.5 (2–11.25)	57 (5.75–195.25)
No increased risk	4 (1–11)	48 (0–238.00)
Very high risk	5 (1–14)	79 (0–373.5)

* BCM (1 January 2010–31 December 2018).

BCM, body composition measurement.

SP who were first seen for MTSS as recruits had fewer days in the care of a healthcare professional but a similar number of contacts to trained SP. Asian SP had fewer contacts over fewer days than all other ethnicities; however, this was based on a very small sample of the population (only 2% of the population was categorised as Black, Asian and minority ethnic (BAME)). Differences were also found in the number of days in care between BCM categories (see online supplemental file 1). However, there was a large amount of missing data, with 38.65% of BCM not reported (table 3).

## Discussion

This is the first large-scale study to report the prevalence of MTSS across the UK Armed Forces. We found MTSS reduced between 2013 and 2018 but continued to affect a large number of SP. Over recent years, each service has implemented changes aiming to reduce musculoskeletal injury. For example, the British Army has introduced new service-issue footwear and a new system of periodised training with a greater emphasis on strength and conditioning and reduced running. With policies and priorities differing across services and between units, it is difficult to quantify which of these changes, if any, may have had an impact on the prevalence of MTSS.

Our study found MTSS to be consistently higher in recruits than trained SP. Overuse injuries requiring medical attention are reported to affect between 15% and 31% of recruits during basic training,^11^ with 82% affecting the lower limbs.^12^ While minimum levels of fitness are required, preparatory training prior to joining the Armed Forces is mainly left to the individual and will vary in quality and quantity. This leaves those who have lower fitness levels at a higher risk of injury.^13^ Fewer years of running experience have been associated with an increased risk of developing MTSS^7^ and may contribute to the high prevalence of MTSS in recruits who may be less experienced runners and less accustomed to high training volumes.

Although more than double that of the general Armed Forces population, the prevalence of MTSS in recruits reported in our study (4.34% (95% CI 4.00 to 4.69 in 2018) was much lower than that of previous studies, for example, 35% of Australian naval recruits reported to have MTSS.^3^ The high numbers reported in the naval study may in part be due to recruits being informed about the syndrome in advance and assured reporting symptoms would not impact their career. Underreporting of musculoskeletal injuries is common within the military, with almost half of the injuries not being reported by SP due to concerns of being put on restricted duties or the potential negative career impact.^8^ It is likely that the different data collection approaches used in the studies account for some of the variation in the prevalence reported, with only those that had received medical attention being included in our study.

The prevalence of MTSS in female recruits and trained SP in our study was higher than in male SP (figure 2). Being female has been reported to be a non-modifiable risk factor for musculoskeletal injury in the Armed Forces.^14^ Female recruits have been reported to be 1.67 times more at risk of calf/shin overuse injury than male recruits.^15^ Possible explanations include altered mechanics during running^16^ and lower bone density in females.^17^

Over a quarter of SP (27%) with MTSS only had one contact with a healthcare professional for MTSS. This may have been due to effective early load management, improvement in symptoms as part of the natural history or a failure to seek further care. The large IQR in the contacts with a healthcare professional (median 4 (IQR 1–12) and days under the care of a healthcare professional 55 (IQR 0–306.5) demonstrates the high variability in the input received for MTSS.

The median length of time under the care of a healthcare professional for MTSS was approximately 2 months, shorter than the reported recovery time of approximately 80 days reported in army recruits^5^ and less than half of the 4–5 months reported in the Dutch Armed Forces.^6^ Differences in recovery times may be partly due to varying definitions of recovery, with our study’s results being based on the duration between the first and last appointment and not complete resolution of symptoms. The outflow from UK regular forces (total number of SP leaving the Armed Forces) may have influenced the results of our study, with 14 698 leaving in the year 2018 alone.^10^ Any SP with MTSS who exits the Armed Forces would no longer be part of this study even if they continued experiencing symptoms.

When comparing healthcare input, female SP received one more contact and 11.5 more days under a healthcare professional than male SP. Although small, there are likely to be multiple factors affecting the amount of healthcare input received. For example, female SP have been reported to have a lower starting fitness level on joining the Armed Forces than male SP.^18^ This may lead them to require more healthcare input to achieve the same required fitness targets.

RM had fewer days under the care of a healthcare professional than all other services. The initial training course at the Commando Training Centre is 32-week long and arguably one of the most demanding of all initial training courses. RM may have higher baseline fitness levels and previous running experience, giving them a good understanding of their training abilities and recovery; however, they may also be less likely to seek medical care than other service branches.

Between 2010 and 2019, 2% of all SP and 3.4% of recruits with MTSS were discharged from the Armed Forces due to a lower limb injury. Many SP with MTSS have concurrent injuries and it is not known whether MTSS or another lower limb injury led to medical discharge. The majority of SP with MTSS were able to continue within the Armed Forces; however, it is not known whether the MTSS led to personnel being downgraded (where SP have been categorised as unfit to complete their occupational role) or if it affected their ability to achieve promotion.

The exact operational and financial cost of MTSS to the Armed Forces was beyond the scope of this study but is likely multifactorial and considerable. Costs attributed to all musculoskeletal injury in the forces over a 10-year period based on lost training days are vast, projected to amount to £329 079 million and the total cost of medical staff reported as £335.169 million.^19^ This does not include costs in relation to diagnosis, treatment or medical overheads. SP who are medically discharged from the forces may also be entitled to claim on the Armed Forces Compensation Scheme, which will compensate for any injury, illness or death caused by service with compensation awarded up to a maximum of £650 000.^20^ In addition to this, there is the payment of military pensions. Although the cost of this to the forces for those discharged in relation to MTSS is not known, when considering all MSK injuries over a 10-year period, the projected cost of pensions alone has been reported to be £665 million^19^ The higher prevalence of MTSS within training units is also likely to have a substantial financial impact on the Armed Forces, with the cost of training recruits at an army training centre £38 000 per recruit.^21^

The main limitation of this study was that the data were not originally created for research purposes. This led to there being large amounts of missing data in some categories, for example, BCM categories, which were reported as the closest available measure to body mass index (associated with the development of MTSS), but could not accurately be reported due to missing data. There was also the need to group some characteristics, for example, BAME categories, where the MTSS data and baseline data were reported differently. The read-code ELLP (representing 8.2% of SP with MTSS) was included in the MTSS data to ensure that all SP with MTSS is accounted for in the data. However, it should be noted that ELLP encompasses several conditions, of which MTSS is the most common, but a small number of SP will be included in the MTSS data that may not have MTSS. The prevalence of MTSS may be higher than that reported, with underreporting of injury being common in the military, with 49% of injuries not being reported in previous military studies.^8^

A strength of our study is the large sample size, as it includes all SP over several years and is the first to report the prevalence of MTSS in the UK Armed Forces. Future research to help determine the impact of policy changes, for example, changes in training systems, would help to understand better the effects of service improvements on MTSS. Understanding why certain populations receive more healthcare input could also lead to better management of those with the syndrome. Currently, there is no effective treatment for individuals with MTSS based on high-quality studies.^22^ Developing an intervention for treating those with MTSS would benefit both the individual and the Armed Forces, enabling them to maintain a fully operational workforce.

## Conclusion

MTSS places a significant burden on the health services within the Armed Forces and although the prevalence of MTSS has reduced across the Armed Forces, it remains high in recruits. As with many overuse conditions, MTSS is more prevalent in women than men, with the highest prevalence in female recruits. There was a large variability in healthcare input received for MTSS between individuals; however, it places a significant burden on both the individual with MTSS and the Armed Forces.

## Supplementary material

10.1136/military-2024-002788 online supplemental file 1

10.1136/military-2024-002788 online supplemental figure 1

## Data Availability

No data are available.
