# Supplementary material for: Prevalence of medial tibial stress syndrome in the British Armed Forces: a population-based study
Source: BMJ Mil Health. 2024 Nov 21;172(1):e002788. doi: 10.1136/military-2024-002788 (PMC12911586; doi:10.1136/military-2024-002788)
Supplement: online supplemental file 1 [file military-172-1-s001.pdf]

Body Composition Measurement

BMI		Waist Circumference		
$kg.m^{-2}$	Classification	Low<br>Men <94 cm<br>Women <80 cm	High<br>Men 94 – 101.9 cm<br>Women 80 – 87.9 cm	Very High<br>Men ≥102 cm<br>Women ≥88 cm
< 18.5	Underweight	Increased Risk		
18.5 – 24.9	Healthy Weight	No Increased Risk		
25.0 – 29.9	Overweight	No Increased Risk	Increased Risk	High Risk
30.0 – 34.9	Obese Class I	Increased Risk	High Risk	Very High Risk
≥ 35.0	Obese Class II and III	Very High Risk		
