# Supplementary material for: Prevalence of medial tibial stress syndrome in the British Armed Forces: a population-based study
Source: BMJ Mil Health. 2024 Nov 21;172(1):e002788. doi: 10.1136/military-2024-002788 (PMC12911586; doi:10.1136/military-2024-002788)
Supplement: online supplemental figure 1 [file military-172-1-s002.pdf]

Read codes

ID	Patient identifier
SQL_Date	Date the MTSS read code was entered
Underlying_code	Read code:<br>• DMSRC194<br>• N21y0<br>• N21y1<br>• 8D58<br>• DMSRC146
Description	Description of the read code:<br>• Exertional lower limb pain (other)<br>• Anterior shin splints<br>• Posterior shin splints<br>• Shin splint<br>• Medial tibial stress syndrome
Practice_name	The practice that the clinician who entered the read code is based
Service	Service the patient belongs to:<br>• Navy<br>• Royal Marines<br>• Army<br>• RAF
FT Trained & Serving against Requirement Mrk	Training status:<br>• 1 = fully trained<br>• 0 = untrained
Sex	Sex of the patient:<br>• Female<br>• Male
BCM_date	Date the BCM recording was taken
BCM_Category	BCM category of the patient – this is the closest recording to the read code date:<br>• NULL = no recording<br>• No increased risk<br>• Increased risk<br>• High risk<br>• Very high risk<br>• Extreme risk
Ethnicity	The ethnicity of the patient:<br>• White Background<br>• Black Caribbean<br>• Mixed Black African and White<br>• Black African<br>• Declined to Declare<br>• White English/Welsh/Scottish/Northern Irish/British<br>• Other Mixed Ethnic Background<br>• Other Asian Background<br>• No Value<br>• Other Ethnic Background<br>• Mixed Black Caribbean and White

	• Any other White background<br>• Asian Indian<br>• Mixed Asian and White<br>• Asian Bangladeshi<br>• Other Black Background<br>• Any Chinese Background<br>• Asian Pakistani<br>• White Gypsy or Irish Traveller<br>• White Irish
Age	Age of the patient at the date the MTSS read code was recorded (in years).
Months_in_service	Months the patient has been in service as at the date the MTSS read code was entered
Medical_discharge	If the patient was medically discharged. If yes then whether MTSS was the principal reason for their discharge or a contributory reason. There can only be one principal reason for a medical discharge but there can be multiple contributory reasons:<br>• No<br>• Principal<br>• Contributory
